# Inflammatory Biomarkers as Prognostic Factors of Acute Deep Vein Thrombosis Following the Total Knee Arthroplasty

**DOI:** 10.3390/medicina58101502

**Published:** 2022-10-21

**Authors:** Răzvan Marian Melinte, Emil Marian Arbănași, Adrian Blesneac, Dan Nicolae Zolog, Réka Kaller, Adrian Vasile Mureșan, Eliza Mihaela Arbănași, Ioana Marta Melinte, Raluca Niculescu, Eliza Russu

**Affiliations:** 1 Department of Orthopedics, Regina Maria Health Network, 540098 Targu Mures, Romania; 2 Department of Orthopedics, Humanitas MedLife Hospital, 400664 Cluj Napoca, Romania; 3 Clinic of Vascular Surgery, Mures County Emergency Hospital, 540136 Targu Mures, Romania; 4 Department of Surgery, George Emil Palade University of Medicine, Pharmacy, Science, and Technology of Targu Mures, 540139 Targu Mures, Romania; 5 Faculty of Pharmacy, George Emil Palade University of Medicine, Pharmacy, Science, and Technology of Targu Mures, 540139 Targu Mures, Romania; 6 Faculty of Medicine, George Emil Palade University of Medicine, Pharmacy, Science, and Technology of Targu Mures, 540139 Targu Mures, Romania; 7 Department of Pathophysiology, George Emil Palade University of Medicine, Pharmacy, Science, and Technology of Targu Mures, 540139 Targu Mures, Romania

**Keywords:** TKA, DVT, MLR, NLR, PLR, SII, SIRI, AISI, inflammatory biomarkers

## Abstract

*Background and objectives*: Deep vein thrombosis (DVT) is one of the most serious post-operative complications in the case of total knee arthroplasty (TKA). This study aims to verify the predictive role of inflammatory biomarkers [monocyte-to-lymphocyte ratio (MLR), neutrophil-to-lymphocyte ratio (NLR), platelets-to-lymphocyte ratio (PLR), systemic inflammatory index (SII), systemic inflammation response index (SIRI), and aggregate index of systemic inflammation (AISI)] in acute DVT following TKA. *Materials and methods*: The present study was designed as an observational, analytical, retrospective cohort study and included all patients over 18 years of age with surgical indications for TKA, admitted to the Department of Orthopedics, Regina Maria Health Network, Targu Mures, Romania, and the Department of Orthopedics, Humanitas MedLife Hospital, Cluj-Napoca, Romania between January 2017 and July 2022. The primary endpoint was the risk of acute DVT following the TKA, and the secondary endpoint was the length of hospital stay, and the outcomes were stratified for the baseline’s optimal MLR, NLR, PLR, SII, SIRI, and AISI cut-off value. *Results*: DVT patients were associated with higher age (*p* = 0.01), higher incidence of cardiac disease [arterial hypertension (*p* = 0.02), atrial fibrillation (*p* = 0.01)], malignancy (*p* = 0.005), as well as risk factors [smoking (*p* = 0.03) and obesity (*p* = 0.02)]. Multivariate analysis showed a high baseline value for all hematological ratios: MLR (OR: 11.06; *p* < 0.001), NLR (OR: 10.15; *p* < 0.001), PLR (OR: 12.31; *p* < 0.001), SII (OR: 18.87; *p* < 0.001), SIRI (OR: 10.86; *p* < 0.001), and AISI (OR: 14.05; *p* < 0.001) was an independent predictor of DVT after TKA for all recruited patients. Moreover, age above 70 (OR: 2.96; *p* = 0.007), AH (OR: 2.93; *p* = 0.02), AF (OR: 2.71; *p* = 0.01), malignancy (OR: 3.98; *p* = 0.002), obesity (OR: 2.34; *p* = 0.04), and tobacco (OR: 2.30; *p* = 0.04) were all independent predictors of DVT risk. *Conclusions*: Higher pre-operative hematological ratios MLR, NLR, PLR, SII, SIRI, and AISI values determined before operations strongly predict acute DVT following TKA. Moreover, age over 70, malignancy, cardiovascular disease, and risk factors such as obesity and tobacco were predictive risk factors for acute DVT.

## 1. Introduction

Total knee arthroplasty (TKA) is one of the most common orthopedic surgeries performed worldwide, addressing severe cases of knee osteoarthritis, and improving patients’ quality of life and their capability to move [1,2,3]. Recently, advancement in operating techniques, as well as the vast variety of prostheses available, have resulted in a significant decrease in post-surgical complications, an increase in success rate, and therefore an improvement in patient quality of life [4,5,6].

Deep vein thrombosis (DVT) is one of the most serious post-operative complications in the case of TKA, leading to pulmonary embolism (PE) and mortality [7,8,9,10]. Furthermore, TKA is associated with a greater incidence of DVT occurrence compared to total hip arthroplasty (THA) [11].

Thrombosis occurs when the key components of Virchow’s triad, including blood circulation stagnation, endothelial injury, and hypercoagulability, are present [12]. Numerous studies have demonstrated the association of inflammatory status with hypercoagulability status [13,14]. Moreover, various inflammatory biomarkers with predictive significance in the diagnosis of post-TKA DVT have been studied in the last ten years [15,16,17]. Interleukin-6 (IL-6) and C-reactive protein (CRP) are two of the most studied inflammatory biomarkers in the prediction of complications following TKA [18,19,20,21], but unfortunately, the results are inconsistent and unsatisfactory, with high sensitivity but low specificity [22,23].

Hematological reports, based on neutrophil, monocyte, lymphocyte, and platelets total number: monocyte-to-lymphocyte ratio (MLR), neutrophil-to-lymphocyte ratio (NLR), platelet-to-lymphocyte ratio (PLR), systemic inflammatory index (SII), systemic inflammation response index (SIRI), and aggregate index of systemic inflammation (AISI), are routine inflammatory biomarkers, easy to use, with low costs. Moreover, the predictive role of these reports has been recently demonstrated in the literature in the case of cardiovascular pathology [24,25,26,27], pulmonary embolism [28,29], chronic kidney disease [30,31,32], the case of COVID-19 patients [33,34,35,36,37,38], as well as in the case of peri-prosthetic infection after total joint arthroplasty [39,40,41].

The risk of DVT following TKA is unpredictable, making therapy for these patients problematic. In modern medicine, the use of diagnostic tools in risk group stratification is crucial since it allows us to establish a treatment plan and prevent thromboembolic events. Furthermore, there is a lack of understanding in the existing literature about the diagnostic methods for DVT risk following joint arthroplasty.

The purpose of this study is to confirm the prognostic significance of hematological ratios (MLR, NLR, PLR, SII, SIRI, and AISI) in the acute DVT following TKA.

## 2. Materials and Methods

### 2.1. Study Design

The present study was designed as an observational, analytical, retrospective cohort case-control study and included all patients over 18 years of age with surgical indications for TKA, admitted to the Department of Orthopedics, Regina Maria Health Network, Targu Mures, Romania, and the Department of Orthopedics, Humanitas MedLife Hospital, Cluj-Napoca, Romania between January 2017 and July 2022. Patients having a history of DVT, patients who required post-operative blood transfusions, and patients who benefitted from bilateral TKA were all excluded.

Patients included in the study were initially divided into two groups depending on the presence of DVT at four weeks named “no-DVT” and “DVT”. The ideal cut-off value for MLR and AISI was used to calculate the DVT risk and length of hospital stay.

### 2.2. Data Collection

Age and gender were obtained from the hospital’s computerized database. The medical history was searched for the following comorbidities: atrial fibrillation (AF), arterial hypertension (AH), chronic heart failure (CHF), ischemic heart disease (IHD), chronic kidney disease (CKD), myocardial infarction (MI), peripheral arterial disease (PAD), type 2 diabetes (T2D), chronic venous insufficiency (CVI), dyslipidemia, obesity (BMI over 30), and malignancy (active cancer). We also gathered information from the patient blood test result (blood urea nitrogen level, cholesterol level, triglyceride level, hematocrit, hemoglobin, glucose level, creatinine level, lymphocyte count, monocyte count, neutrophil count, platelet count, potassium level, sodium level, serum calcium, glomerular rate filtration, and serum albumin).

### 2.3. Systemic Inflammatory Markers

The systemic inflammation index was determined from the first blood test result. The ratio was calculated using the equations:

- MLR = total number of monocytes/total number of lymphocytes
- NLR = total number of neutrophils/total number of lymphocytes
- PLR = total number of platelets/total number of lymphocytes
- SII = (total number of neutrophils × total number of platelets)/total number of lymphocytes
- SIRI = (total number of monocytes × total number of platelets)/total number of lymphocytes
- AISI = (total number of neutrophils × total number of monocytes × total number of platelets)/total number of lymphocytes

### 2.4. Knee Osteoarthritis Severity

Regarding the severity of knee osteoarthritis, we used one of the most frequently applied classifications based on pre-operative radiography, the Kellgren–Lawrence classification, which ranges from grade 0 to 4, depending on the progression and severity of osteoarthritis [42]. Furthermore, patients with an injury below Kellgren–Lawrance grade 3 were excluded from the research.

### 2.5. Surgical Technique

All patients had the same surgical method, which was undertaken by the same specialists. The Zimmer Biomet NexGen LPS implant was used for the procedure, and tranexamic acid was injected shortly after the joint capsule was closed. All patients were given intravenous antibiotics and low molecular weight heparin within the first 72 h of surgery. Anti-embolism stockings were used on all patients to avoid DVT. Furthermore, for postoperative days 3 to 14, an anticoagulant (rivaroxaban, Xarelto^®^, 10 mg, Bayer AG, Leverkusen, Germany) was administered. Lastly, all patients began physical therapy on the first day post-surgery.

### 2.6. Study Outcomes

The primary endpoint was the risk of acute DVT, and the secondary endpoint was the length of hospital stay. Outcomes were stratified for the baseline’s optimal MLR, NLR, PLR, SII, SIRI, and AISI cut-off value.

### 2.7. Follow-up Strategy

Post-operatively, all patients were evaluated with a doppler ultrasonography, to identify DVT before being discharged and again at four weeks. During the hospitalization, and following the TKA, none of the 273 participants in the study had acute DVT.

### 2.8. Statistical Analysis

SPSS for Mac OS version 28.0.1.0 was used for statistical analysis (SPSS, Inc., Chicago, IL, USA). Chi-square tests were used to assess the associations of hematological ratios with category factors, while t-Student or Mann–Whitney tests were used to assess differences in continuous variables. To analyze the predictive power and to establish the cut-off values of hematological ratios, the receiver operating characteristic (ROC) curve analysis was utilized. The ROC curve analysis was used to determine the appropriate MLR, NLR, PLR, SII, SIRI, and AISI cut-off values based on the Youden index (Youden Index = Sensitivity + Specificity − 1, ranging from 0 to 1). To identify independent predictors of DVT risk, a multivariate logistic regression analysis using variables with *p* < 0.1 was undertaken.

## 3. Results

During the study period, 339 patients were hospitalized, with 28 requiring post-operative blood transfusions, 22 having a history of DVT, and 16 having bilateral TKA. Throughout the research procedure, 273 patients were enrolled in the study. At four weeks, 28 patients (10.25%) had a DVT diagnosis confirmed by ultrasonography, although there was no indication of pulmonary embolism. Of the 28 patients with DVT, 22 had DVT below the knee, 3 had mild edema at the level of the afflicted limb, and the remaining 19 had no symptoms. The six patients who presented with DVT above the knee had significant edema and functional impotence at the afflicted leg level and required anticoagulant medication.

In terms of demographics, confirmed DVT patients were older (*p* = 0.0006). Additionally, cardiovascular pathologies [AH (*p* = 0.02), AF (*p* = 0.01)], malignancy (*p* = 0.005), as well as risk factors [smoking (*p* = 0.03) and obesity (*p* = 0.02)] were more prevalent in the DVT group. Moreover, in the case of laboratory analyses, there was a low level of hemoglobin (*p* = 0.02), hematocrit (*p* = 0.03), the total number of lymphocytes (*p* < 0.0001), and albumin (*p* = 0.002), as well as an increased level of the total number of neutrophils (*p* = 0.0001), monocytes (*p* = 0.001), platelets (*p* = 0.0007), BUN (*p* = 0.02), creatinine (*p* < 0.0001), potassium (*p* = 0.03), and hematological reports (for all *p* < 0.0001), as seen in Table 1.

**Figure 1:**
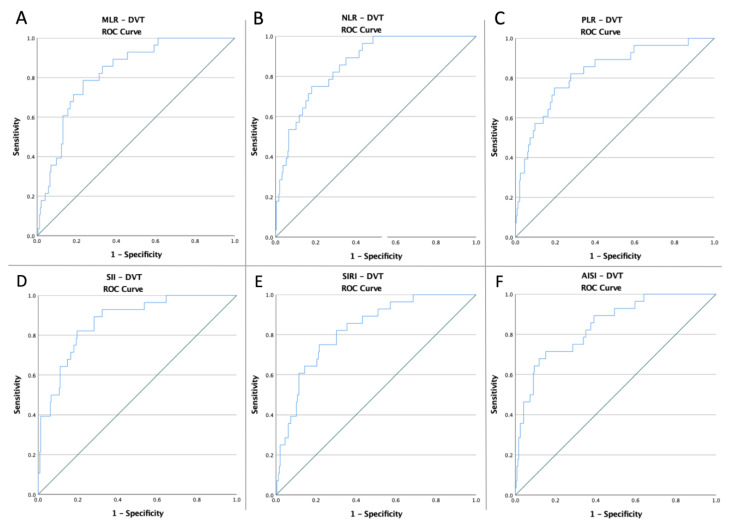
ROC curve analysis concerning DVT risk (**A**) for the MLR (AUC: 0.825; *p* < 0.0001), (**B**) for the NLR (AUC: 0.862; *p* < 0.0001), (**C**) for the PLR (AUC: 0.829; *p* < 0.0001), (**D**) for the SII (AUC: 0.870; *p* < 0.0001), (**E**) for the SIRI (AUC: 0.824; *p* < 0.0001), and (**F**) for the AISI (AUC: 0.842; *p* < 0.0001).

The ROC curves of MLR, NLR, PLR, SII, SIRI, and AISI were created to determine whether the baseline of these markers was predictive of acute DVT following the TKA (Figure 1). The optimal cut-off value obtained from Youden’s index, areas under the curve (AUC), and the predictive accuracy of the markers are listed in Table 2.

The DVT risk and length of hospital stay were further analyzed after dividing the patients into paired groups, according to the optimal cut-off value of inflammatory biomarkers. Regarding the hospitalization time, there was a longer inpatient stay in the high-NLR (*p* = 0.01), high-SIRI (*p* = 0.02), and high-AISI group (*p* = 0.0004). Moreover, there was a higher incidence of DVT risk for all the hematological ratios, as seen in Table 3.

A multivariate analysis was used to determine the association between the hematological ratios, the underlying risk factors, and DVT. A high baseline value of all hematological ratios was a strong independent predictor of DVT (for all *p* < 0.0001). Moreover, as indicated in Table 4, age above 70 (OR: 2.96; *p* = 0.007), AH (OR: 2.93; *p* = 0.02), AF (OR: 2.71; *p* = 0.01), malignancy (OR: 3.98; *p* = 0.002), obesity (OR: 2.34; *p* = 0.04), and tobacco (OR: 2.30; *p* = 0.04) were all independent predictors of DVT risk.

**Figure 2:**
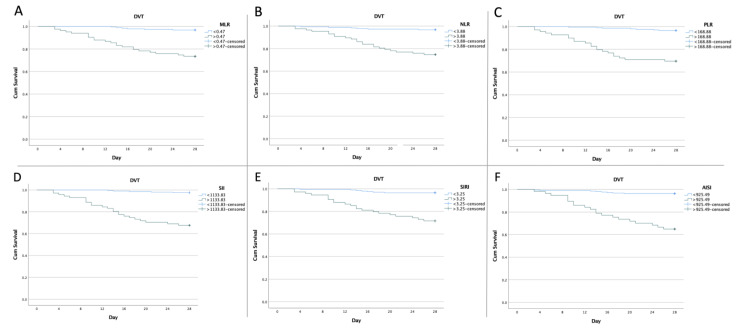
Kaplan–Meier curves showing DVT risk (**A**) according to MLR optimal cut off value (*p* <0.001; log-rank *p*), (**B**) according to NLR optimal cut-off value (*p* < 0.001; log-rank *p*), (**C**) according to PLR optimal cut-off value (*p* < 0.001; log-rank *p*), (**D**) according to SII optimal cut-off value (*p* < 0.001; log-rank *p*), (**E**) according to SIRI optimal cut-off value (*p* < 0.001; log-rank *p*), and (**F**) according to AISI optimal cut-off value (*p* < 0.001; log-rank *p*).

The Kaplan–Meier plot for the DVT risk in the first 4 weeks post-operation based on the optimal cut-off value of the hematologic ratios is shown in Figure 2.

## 4. Discussion

The primary outcome of this research is that pre-operative hematological inflammatory markers have a strong predictive role in the risk of DVT incidence following TKA. We further confirm that cardiovascular disease (AH and AF), older age, malignancy, as well as smoking and obesity all predict DVT risk, as shown in Table 4. To the best of our knowledge, this is the first study to evaluate the predictive role of all hematological biomarkers and risk of DVT following TKA.

The incidence of acute DVT in patients undergoing TKA has been identified to be associated with elderly patients, a history of malignancy, and cardiovascular pathology [43,44,45]. TKA presents all three factors of the Virchow triad. Venous stasis occurs both intra-operatively, due to surgical immobilization and the use of a tourniquet, and shortly post-operatively, due to poor mobilization [46,47]. Endothelial damage is also unavoidable in major surgery such as TKA [47,48]. Moreover, the operational act causes local inflammation as well as a systemic inflammatory response that promotes a hypercoagulable condition [49,50,51].

Another risk factor for DVT following TKA is the use of tranexamic acid, which, although reducing the need for a post-operative blood transfusion by up to 39%, has been shown to have a predictive role in the occurrence of DVT in articles by Myers et al. [52], Henry et al. [53], and Ng et al. [54].

The predictive role of inflammatory biomarkers was analyzed and also demonstrated in the case of periprosthetic joint infection [39,40,41,55,56,57]. In the systemic review published by Festa et al. [39], it was demonstrated that high values of MLR, NLR, and PLR are associated with the detection of hip and knee periprosthetic infection.

In the work published by Yao et al., it was demonstrated that elderly patients (OR: 1.05; 95% CI: 1.02–1.07; *p* < 0.001) as well as high pre-operative and post-operative values of NLR (OR: 1.11; 95% CI: 1.01–1.23; *p* = 0.025 and OR: 1.20; 95% CI: 1.12–1.30; *p* < 0.001) are predictive factors for acute DVT after total joint arthroplasty [58]. Moreover, Seo et al. demonstrated that the pre-operative values of NLR > 1.9 (OR: 1.95; 95% CI: 1.16–3.31; *p* = 0.01) are independent predictors of venous thromboembolism in 264 patients undergoing TKA [59].

According to our results, the high pre-operative values of hematological reports: MLR (OR: 11.06; *p* < 0.001), NLR (OR: 10.15; *p* < 0.001), PLR (OR: 12.31; *p* < 0.001), SII (OR: 18.87; *p* < 0.001), SIRI (OR: 10.86; *p* < 0.001), and AISI (OR: 14.05; *p* < 0.001) are strong independent factors predicting the risk of acute DVT following TKA. In the univariate analysis, high NLR, SII, and AISI values were also associated with a prolonged inpatient stay, as shown in Table 3.

Hematological ratios (MLR, NLR, PLR, SII, SIRI, and AISI) are measures of acute myeloid-driven innate immune responses reported to chronic, lymphocyte-driven, immunological memory reflected by lymphocyte numbers. An increased hematological ratio may reflect an immunological imbalance between a potential ongoing clinical or sub-clinical acute inflammation and an impaired immune defense. The importance of these hematological indicators in predicting coagulopathy risk and thromboembolic risk was widely researched [24,26,29].

Although this study included TKA patients from two private hospitals over a five-year period and had significant results in terms of the high level of sensitivity and specificity of the investigated inflammatory biomarkers [MLR (78.6% Sensitivity and 76.7% Specificity), NLR (78.6% Sensitivity and 73.5% Specificity), PLR (75% Sensitivity and 80.4% Specificity), SII (82.1% Sensitivity and 80.4% Specificity), SIRI (75% Sensitivity and 78.4% Specificity), and AISI (71.4% Sensitivity and 84.9% Specificity)] in the prediction of acute DVT, it has certain limitations. Firstly, we must consider the retrospective design of the study. Additionally, tranexamic acid was used for intra-articular injections in all included patients. Because this research was done using data from two private medical centers, we may expect the patients to have better health status.

## 5. Conclusions

Our data revealed that higher pre-operative hematological ratios MLR, NLR, PLR, SII, SIRI, and AISI values highly predict acute DVT following TKA. Moreover, during the studied period, age above 70, malignancy, cardiovascular disease, and risk factors such as obesity and tobacco were predictive risk factors for acute DVT. Given their accessibility and low cost, these ratios can be used for pre-operative risk group stratification, for better patient management regarding the DVT enhancement of thromboprophylaxis and for the development of predictive patterns.

## Figures and Tables

**Table 1:** The baseline characteristics data of all patients and divided according to the DVT risk.

Variables	All Patients n = 273	No-DVT n = 245	DVT n = 28	*p* Value (OR; CI 95%)
Age mean ± SD (min–max)	66.36 ± 6.91 (51–82)	65.26 ± 6.81 (51–82)	68.71 ± 6.55 (51–77)	0.006
Male/Female sex no. (%)	122 (44.69%) 151 (55.31%)	110 (44.90%) 135 (55.10%)	12 (42.86%) 16 (57.14%)	0.83 (0.92; 0.41–2.02)
**Comorbidities and Risk Factors**				
AH, no. (%)	158 (57.87%)	136 (55.51%)	22 (78.57%)	0.02 (2.93; 1.15–7.50)
IHD, no. (%)	134 (49.08%)	119 (48.57%)	15 (53.57%)	0.61 (1.22; 0.55–2.67)
AF, no. (%)	65 (23.80%)	53 (21.63%)	12 (42.85%)	0.01 (2.71; 1.21–6.09)
CHF, no. (%)	101 (36.99%)	90 (36.73%)	11 (39.28%)	0.79 (1.11; 0.49–2.48)
MI, no. (%)	55 (20.14%)	46 (18.77%)	9 (32.14%)	0.10 (2.04; 0.87–4.82)
T2D, no. (%)	99 (36.23%)	87 (35.51%)	12 (42.85%)	0.44 (1.36; 0.61–3.009)
CKD, no. (%)	21 (7.69%)	19 (7.75%)	2 (7.14%)	0.90 (0.91; 0.20–4.15)
Malignancy, no. (%)	20 (7.32%)	14 (5.71%)	6 (21.42%)	0.005 (4.50; 1.57–12.88)
CVI, no. (%)	82 (30.03%)	72 (29.38%)	10 (35.71%)	0.49 (1.33; 0.58–3.03)
Tobacco, no. (%)	80 (29.30%)	67 (27.34%)	13 (46.42%)	0.03 (2.30; 1.04–5.09)
Obesity, no. (%)	61 (22.34%)	50 (20.40%)	11 (39.28%)	0.02 (2.52; 1.11–5.72)
Dyslipidemia, no. (%)	66 (24.17%)	55 (22.44%)	11 (39.28%)	0.053 (2.23; 0.98–5.05)
**Laboratory Data**				
Hemoglobin g/dL median (Q1–Q3)	14.4 (12.8–15.3)	14.47 (12.9–15.31)	13.2 (12.27–15.15)	0.02
Hematocrit % median (Q1–Q3)	41.2 (38.2–43.45)	41.23 (38.5–43.45)	39.1 (36.07–42.58)	0.03
Neutrophils ×10^3^/μL median (Q1–Q3)	5.86 (4.47–7.6)	5.68 (4.39–7.5)	7.42 (6.37–9.61)	0.0001
Lymphocytes ×10^3^/μL median (Q1–Q3)	1.82 (1.46–2.4)	1.9 (1.53–2.44)	1.27 (1.02–1.79)	<0.0001
Monocyte ×10^3^/μL median (Q1–Q3)	0.63 (0.46–0.89)	0.62 (0.46–0.86)	0.83 (0.59–1.22)	0.001
PLT ×10^3^/μL median (Q1–Q3)	239 (202–301.45)	235.4 (200.2–297.8)	285.9 (240.25–342.75)	0.0007
Glucose mg/dL median (Q1–Q3)	118 (96–142)	117 (96–143)	120.5 (95.25–139.25)	0.43
Cholesterol mg/dL median (Q1–Q3)	129.1 (104.7–163.7)	130.8 (105.6–164)	121.3 (94.12–144.47)	0.11
Triglyceride mg/dL median (Q1–Q3)	123.7 (90.8–176)	123 (90.8–176)	136.8 (101.87–173.4)	0.36
BUN mg/dL median (Q1–Q3)	40 (31.9–49.2)	39.4 (31.8–48.6)	46 (37.77–55.22)	0.02
Creatinine mg/dL median (Q1–Q3)	0.89 (0.75–1.04)	0.88 (0.73–1.02)	1.02 (0.90–1.24)	<0.0001
GFR (mL/min/1.73 M^2^) median (Q1–Q3)	86.15 (75.11–89.2)	86.15 (75.11–88)	86.15 (75.83–98.5)	0.22
Serum albumin mg/dL median (Q1–Q3)	3.44 (2.9–3.96)	3.55 (2.93–4)	3 (2.69–3.62)	0.002
Serum calcium mg/dL median (Q1–Q3)	8.55 (8.13–9.19)	8.58 (8.09–9.23)	8.43 (8.36–8.71)	0.37
Potassium mmol/L median (Q1–Q3)	4.58 (4.1–5.31)	4.56 (4.08–5.24)	4.95 (4.44–5.74)	0.03
Sodium mmol/L median (Q1–Q3)	140 (139–141)	140 (139–141)	140 (139–141)	0.47
MLR, median (Q1–Q3)	0.34 (0.24–0.52)	0.32 (0.23–0.46)	0.60 (0.48–0.77)	<0.0001
NLR, median (Q1–Q3)	3.12 (2.34–4.18)	2.97 (2.25–3.92)	5.71 (4.16–6.75)	<0.0001
PLR, median (Q1–Q3)	129.81 (102.62–169.10)	123.8 (100.33–160.60)	209.55 (166.46–289.81)	<0.0001
SII, median (Q1–Q3)	717.95 (531.61–1144.33)	686.82 (514.28–1058.05)	1534.73 (1168.03–2251.45)	<0.0001
SIRI, median (Q1–Q3)	1.96 (1.28–3.40)	1.86 (1.19–2.85)	4.63 (3.10–6.99)	<0.0001
AISI, median (Q1–Q3)	493.85 (272.82–849.03)	449.32 (250.29–772.92)	1360.18 (701.71–2305.76)	<0.0001
**Outcomes**				
DVT, no. (%)	28 (10.25%)	-	28 (10.25%)	<0.0001
Length of hospital stay, median (Q1–Q3)	8 (7–10)	8 (7–10)	8.5 (7.75–10.25)	0.15

AISI = aggregate index of systemic inflammation; AF = atrial fibrillation; AH = arterial hypertension; BUN = blood urea nitrogen; CHF = chronic heart failure; CKD = chronic kidney disease; CVI = chronic venous insufficiency; DVT = deep vein thrombosis; GFR = glomerular filtration rate; IHD = ischemic heart disease; MI = myocardial infarction; MLR = monocyte to lymphocyte ratio; NLR = neutrophil to lymphocyte ratio; PLR = platelets to lymphocyte ratio; PLT = total platelet count; SII = systemic inflammatory index; SIRI = systemic inflammation response index; T2D = type 2 diabetes.

**Table 2:** AUC of the ROC curve, 95% confidence interval, sensitivity, and specificity of the pre-operative inflammatory markers.

Variables	Cut-Off	AUC	Std. Error	95% CI	Sensitivity	Specificity	*p* Value
DVT							
**MLR**	0.47	0.825	0.035	0.757–0.894	78.6%	76.7%	<0.0001
**NLR**	3.88	0.862	0.030	0.802–0.922	78.6%	73.5%	<0.0001
**PLR**	168.88	0.829	0.042	0.747–0.911	75%	80.4%	<0.0001
**SII**	1133.83	0.870	0.032	0.806–0.933	82.1%	80.4%	<0.0001
**SIRI**	3.25	0.824	0.037	0.751–0.897	75%	78.4%	<0.0001
**AISI**	925.49	0.842	0.038	0.767–0.916	71.4%	84.9%	<0.0001

AISI = aggregate index of systemic inflammation; AUC = area under curve; DVT = deep vein thrombosis; MLR = monocyte to lymphocyte ratio; NLR = neutrophil to lymphocyte ratio; PLR = platelets to lymphocyte ratio; SII = systemic inflammatory index; SIRI = systemic inflammation response index; Std. = standard.

**Table 3:** Univariate analysis of hematological ratios and length of hospital stay and DVT risk.

Variables	Length of Hospital Stay	DVT
**Low-MLR vs. high-MLR**	8.38 ± 1.95 vs. 8.61 ± 2.28 *p* = 0.33	6/190 (3.15%) vs. 22/83 (26.50%) *p* < 0.0001 OR:11.06 CI: (4.28–28.54)
**Low-NLR vs. high-NLR**	8.27 ± 2.006 vs. 8.83 ± 2.13 *p* = 0.01	6/186 (3.22%) vs. 22/87 (25.28%) *p* < 0.0001 OR:10.15 CI: (3.94–26.15)
**Low-PLR vs. high-PLR**	8.39 ± 1.99 vs. 8.62 ± 2.24 *p* = 0.28	7/204 (3.43%) vs. 21/69 (30.43%) *p* < 0.0001 OR:12.31 CI: (4.94–30.64)
**Low-SII vs. high-SII**	8.35 ± 1.97 vs. 8.73 ± 2.28 *p* = 0.13	5/202 (2.47%) vs. 23/71 (32.39%) *p* < 0.0001 OR:14.6 CI: (3.02–70.60)
**Low-SIRI vs. high-SIRI**	8.26 ± 1.95 vs. 8.95 ± 2.26 *p* = 0.02	7/199 (3.51%) vs. 21/74 (28.37%) *p* < 0.0001 OR:13.14 CI: (5.32–32.43)
**Low-AISI vs. high-AISI**	8.21 ± 1.94 vs. 9.36 ± 2.25 *p* = 0.0004	8/216 (3.70%) vs. 20/57 (35.08%) *p* < 0.0001 OR:14.05 CI: (5.76–34.27)

AISI = aggregate index of systemic inflammation; DVT = deep vein thrombosis; MLR = monocyte to lymphocyte ratio; NLR = neutrophil to lymphocyte ratio; PLR = platelets to lymphocyte ratio; SII = systemic inflammatory index; SIRI = systemic inflammation response index.

**Table 4:** Multivariate analysis for predictors of DVT.

Variables	DVT		
	OR	95% CI	*p* Value
>70 years	2.96	1.33–6.57	0.007
AH	2.93	1.15–7.50	0.02
AF	2.71	1.21–6.05	0.01
Malignancy	3.98	1.68–9.43	0.002
Obesity	2.34	1.03–5.30	0.04
Tobacco	2.30	1.04–5.09	0.04
HIGH-MLR	11.06	4.28–28.54	<0.001
high-NLR	10.15	3.94–26.15	<0.001
HIGH-PLR	12.31	4.94–30.64	<0.001
high-SII	18.87	6.82–52.21	<0.001
high-SIRI	10.86	4.38–26.94	<0.001
high-AISI	14.05	5.76–34.27	<0.001

AISI = aggregate index of systemic inflammation; AF = atrial fibrillation; AH = arterial hypertension; DVT = deep vein thrombosis; MLR = monocyte to lymphocyte ratio; NLR = neutrophil to lymphocyte ratio; PLR = platelets to lymphocyte ratio; SII = systemic inflammatory index; SIRI = systemic inflammation response index.

## Data Availability

Not applicable.
